# Inflammation-induced leg length discrepancy in children: from molecular mechanisms to clinical implications

**DOI:** 10.3389/fmed.2025.1542822

**Published:** 2025-05-20

**Authors:** Tim R. J. Aeppli, Zhengpei Zhang, Yunhan Zhao, Farasat Zaman, Lars Sävendahl

**Affiliations:** ^1^Division of Pediatric Endocrinology, Department of Women’s and Children’s Health, Karolinska Institutet, Stockholm, Sweden; ^2^Pediatric Endocrinology Unit, Karolinska University Hospital, Stockholm, Sweden

**Keywords:** bone growth, cytokines, growth plate, inflammation, leg length difference, leg length discrepancy

## Abstract

Leg length discrepancy (LLD) refers to a condition where the lower limbs are of unequal length, which can result from various underlying causes. Inflammatory conditions in children, such as monoarticular, pauciarticular or polyarticular juvenile idiopathic arthritis (JIA), can lead to the development of LLD when predominantly affecting one leg. To date, no review has addressed inflammation-induced LLD. Depending on the localization of the inflammation and age of onset, bone growth can be either locally retarded or accelerated in the affected leg. The resulting LLD can range from mild forms, where treatment is not necessary, to severe forms, leading to premature growth plate fusion and/or an LLD of 5 cm or more. The overall aim of this review is to provide an overview of inflammation-induced LLD and to discuss the possible underlying mechanisms at the growth plate level. In addition, this review offers guidance regarding the natural course of the disease and explores potential new treatment strategies for patients with inflammation-induced LLD.

## 1 Introduction

Leg length discrepancy (LLD) is defined as a difference in length between the two legs (1). It can be either acquired or congenital and affects approximately one-third of the population when defined as an LLD exceeding 1 cm (2, 3). Acquired LLD can develop due to the growth-modulating effects of local underlying conditions such as infection, trauma, or inflammation. These factors may either reduce or stimulate the growth of one leg, eventually leading to LLD (4). This review aims to fill a gap in the existing literature by providing an overview of inflammation-induced LLD and its underlying mechanisms, including effects at the growth plate level.

Longitudinal bone growth occurs at the growth plate, a thin layer of cartilage located between the epiphysis and metaphysis of most long bones (5). The process by which the embryonic cartilaginous model of long bones is replaced by calcified bone is called endochondral ossification (6). In humans and other mammals, the growth plate is highly organized and composed of three different zones: resting, proliferative, and hypertrophic zones (7, 8). Chondrocytes are recruited from the resting zone to the hypertrophic zone, where they proliferate and eventually undergo hypertrophy and increase their volume dramatically. At the same time, they also secrete extracellular matrix, which thereafter becomes mineralized. Matrix secretion between chondrocytes and chondrocyte proliferation causes the elongation of long bones. The hypertrophic chondrocytes then allow the invasion of blood vessels and other bone cell precursors, leading to the remodeling of the hypertrophic zone cartilage into bone (6, 7). The multi-step process of longitudinal bone growth is regulated by various signaling pathways such as the Indian hedgehog (Ihh), the parathyroid-related protein (PTHrP), and the growth hormone (GH)-insulin-like growth factor 1 (IGF-1) axis. In addition to local factors, chondrocytes also respond to external cues such as mechanical loading, nutrition, and inflammation that may affect bone elongation (9).

During childhood, longitudinal bone growth can be either suppressed or accelerated by chronic inflammatory conditions (10–13). If the inflammation is systemic, bone growth is often equally affected in both legs. In contrast, if the inflammatory process predominantly affects only one leg, the growth of that leg can be either promoted or inhibited, leading to the development of LLD (10, 11).

## 2 Search strategy and selection criteria

**Figure 1:**
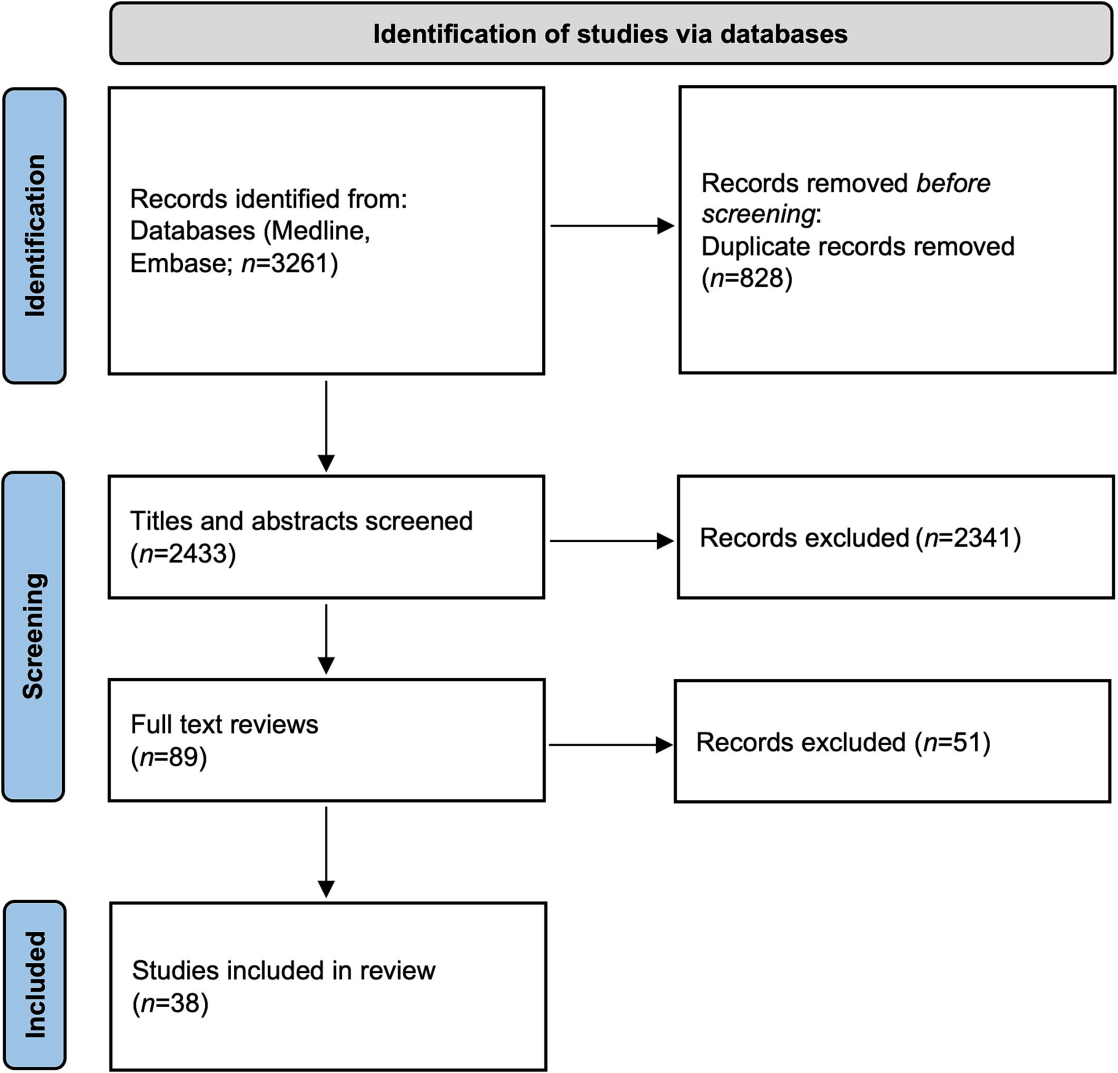
Flow diagram of included studies in this review.

The literature search was conducted in October 2021 and updated in March 2024. References supporting the core sections 4 and 5, which addresses LLD and inflammation-induced LLD, respectively, were identified through searches of Medline (Ovid) and Embase using the search terms “leg length discrepancy,” “leg length inequality,” “leg length difference,” and “limb length” in combination with “inflammation” and “arthritis.” The identified records were then imported into Covidence software (Covidence systematic review software, Veritas Health Innovation, Melbourne, Australia)^1^. Studies were included if they met the following criteria: (1) the paper was published in English and (2) the studied population consisted of pediatric patients. Abstract and title screening was performed independently by two investigators (T. A. and Z. Z.), who also reviewed the full texts of potentially relevant studies. Any discrepancies in the selection of papers were resolved through discussion (T. A. and Z. Z.). A flow diagram of studies included in this review is provided in Figure 1. The references for section 3 (Effects of chronic inflammation on the growth plate level) were identified based on the authors’ knowledge (one of the key fields of expertise) and searches of Medline and Embase using the search terms “growth plate” and “inflammation” or “arthritis.” The final reference list was generated based on originality and relevance to the broad scope of this review.

## 3 Effects of chronic inflammation at the growth plate level

Bone growth occurs at the growth plate level and is a result of the cartilage being replaced with bone tissue (14). Growth plate chondrocytes hereby drive the growth of skeletal elements and also later form a scaffold, which is thereafter mineralized (14). The growth plate is highly organized and composed of three different zones: the resting zone, the proliferative zone, and the hypertrophic zone (7, 8). The resting zone chondrocytes serve as reserve cells and proliferate slowly, providing a continuous supply of chondrocytes for the whole growth plate. In addition, they have self-renewal capacity, can form columns of chondrocytes (15, 16), and play a crucial role in organizing and coordinating the proliferative zone (8). Chondrocytes in the proliferative zone slightly increase in volume and number, exhibit a higher proliferation rate, and start to create and organize into columns (17). In the hypertrophic zone, chondrocytes increase their volume dramatically and increase the production of collagen type X (18). At the same time, they also secrete the extracellular matrix, which subsequently becomes mineralized and produces vascular endothelial growth factor (VEGF), which promotes vascular invasion (19). The cartilage matrix provides the foundation for osteoblast invasion and, together with blood vessels, produces a true bone matrix, leading to a remodeling of the hypertrophic zone cartilage into bone (6, 7). The fate of hypertrophic chondrocytes remains unclear; however, some studies suggest autophagy (20), transdifferentiation (21), or apoptosis (22).

In chronic inflammatory conditions, immune cells are activated, leading to the production of cytokines, interleukins, chemokines, and interferons (10). The presence of the aforementioned may lead to a disturbance within the growth plate, thereby affecting longitudinal bone growth (23). The important cytokines that are upregulated in conditions of chronic inflammation are tumor necrosis factor *α* (TNFα), interleukin-6 (IL-6), and interleukin-1*β* (IL1-β) (24). The cytokines can also act individually or in synergy to suppress longitudinal bone growth, as shown in *ex vivo* studies (11, 24, 25) as well as *in vivo* animal studies (23).

**Figure 2:**
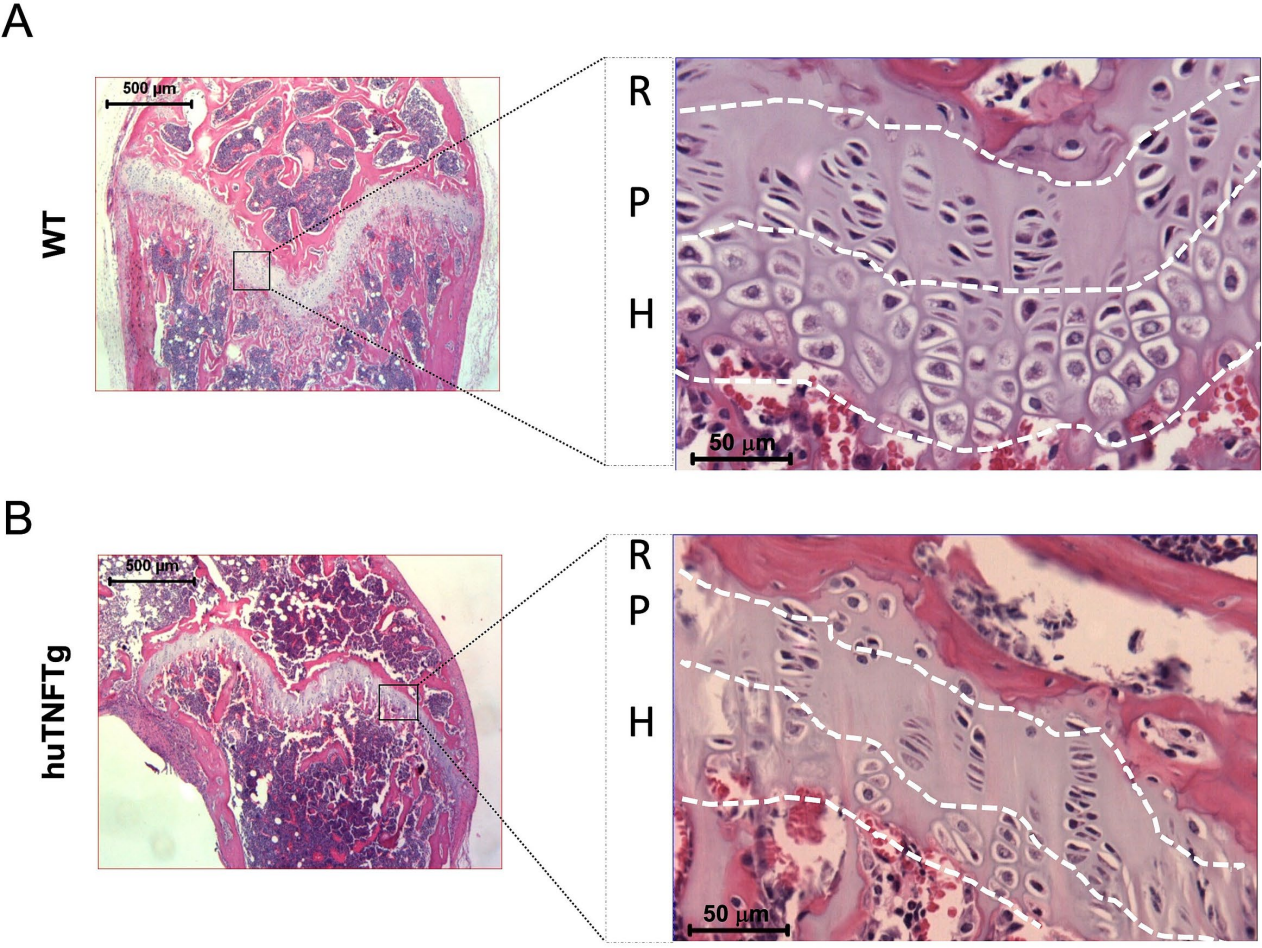
Growth plate morphology of wild type (WT) and huTNFTg mice. Representative hematoxylin–eosin stained femur growth plate sections in **(A)** wildtype and **(B)** huTNFTg mice at × 5 (left) and × 20 magnification (right), indicating the resting (R), proliferative (P), and hypertrophic (H) zone.

TNFα exerts its growth suppressive effect by acting both locally at the growth plate level and systemically at the pituitary level, suppressing the GH/IGF-1 axis (26). Locally, TNFα has been reported to increase chondrocyte apoptosis and decrease chondrocyte proliferation and hypertrophy (11, 25, 27). Furthermore, it also reduces the synthesis of important cartilage matrix components (27). In a recent study, femur bone length and growth plate height were found to be significantly decreased in human TNFα-overexpressing mice (huTNFTg) when compared to healthy controls (23). In Figure 2, we present representative growth plate images of wild-type and huTNFTg mice illustrating the aforementioned differences in growth plate height. Furthermore, huTNFTg mice showed decreased chondrocyte hypertrophy, suppressed Indian hedgehog expression, and disorganized chondrocyte columns (Figure 2). In addition, increased apoptosis was noted in huTNFTg mice, as assessed by the expression of caspase-3 (28). If TNFα leads to increased apoptosis in resting zone chondrocytes, a decreased supply of chondrocytes will be provided to the growth plate, compromising longitudinal bone growth.

Another important inflammatory cytokine that has been shown to have a local effect at the growth plate level is IL-6. IL-6 has been described to inhibit early chondrocyte differentiation, cartilaginous nodule formation *in vitro*, type II and X collagen expression, and aggrecan expression (29). Furthermore, when fetal rat metatarsal bones were cultured *ex vivo* with IL-6 in combination with its soluble receptor, IL-6 Rα, bone growth was found to be decreased (30).

In addition to the growth-inhibitory effect of individual cytokines, a synergistic effect has been described when two or more cytokines are combined (25). In an *ex vivo* culture model, fetal rat metatarsal bones were co-treated with TNFα and IL1-*β*, and longitudinal bone growth was then suppressed to a much larger degree compared to when the bones were exposed to TNFα or IL1-*β* separately (25). In this study, the concentration of the respective cytokine was shown to be a crucial factor, as the negative effects of TNFα and IL1-β alone on chondrocytes can only be observed at higher concentrations (30). Interestingly, both TNFα and IL1-β are also produced locally by growth plate chondrocytes, indicating a critical role in the physiological regulation of bone growth.

The pathophysiology of the growth-promoting effect of cytokines remains unclear. Hyperemia due to the juxtaposed growth plates has been discussed to be a mechanism for overgrowth, although a scientific explanation does not yet exist for this proposed notion (11). Another possible mechanism discussed is that TNFα may induce neo-vascularization *in vivo*, therefore stimulating growth plate vascular invasion (31). Furthermore, IL1-*β* and TNFα have also been shown to increase DNA synthesis in growth plate chondrocytes *in vitro*, which may be a possible explanation for a growth stimulatory effect (11). Interestingly, Mårtensson et al. (25) demonstrated using an *in vitro* rat metatarsal model that TNFα and IL1-β have a stimulatory effect on bone growth at lower concentrations compared to an inhibitory effect at higher concentrations. In line with the aforementioned dose–response effect, there is also a study in humans (32), where TNFα and IL-1β levels in children with different subforms of juvenile idiopathic arthritis (JIA) were assessed. Interestingly, patients with a systemic disease exhibited the highest values of soluble interleukin-2-receptor (sIL-2R), soluble tumor necrosis factor receptor (sTNFR), IL-6, and c-reactive protein (CRP) in comparison to polyarticular or pauciarticular arthritis. Depending on the subtype of JIA, TNFα and IL-1β levels were variably elevated, suggesting that the concentration of various cytokines may determine whether inflammation will promote or suppress bone growth. Furthermore, some studies also report an age-dependent effect of inflammation on growth, leading to a growth-stimulatory effect in younger children and a growth-suppressive effect in older children above 9 years of age (33, 34). However, studies for the reasoning behind this age-dependent difference are lacking. One can speculate that sex steroids may play a role, as children usually enter puberty around that time, leading to the production of estrogen in girls and testosterone in boys (35, 36).

## 4 Leg length discrepancy: definition, epidemiology, and etiology

**Figure 3:**
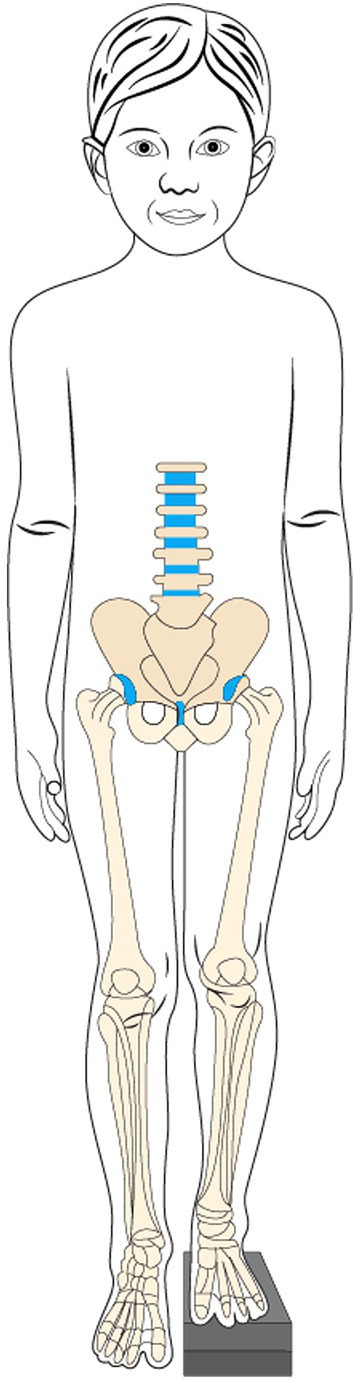
Illustrative figure of leg length discrepancy with a shorter left limb. A level pelvis is achieved by a wooden block.

To assess and quantify leg length in children, long leg radiographs are performed and serve as an important diagnostic tool (37). Leg length can be measured manually or, for example, by artificial intelligence (AI)-based algorithm measurements, as recently reported (38). LLD can be classified into functional and anatomical types (39). Anatomical LLD is characterized by a difference in bone length of the thigh and/or legs, whereas functional LLD occurs when the length of the bones is the same, but there is a joint and/or soft-tissue abnormality that leads to a difference in leg length (39, 40). In this review, we will only focus on anatomical LLD. An illustrative figure of leg length discrepancy where a wooden block is used to achieve a level pelvis, allowing for the measurement of the extent of LLD, is presented in Figure 3. The prevalence of LLD varies depending on the definition. Studies from the US and Sweden have shown that approximately one-third of the population has an LLD greater than 1 cm (2, 3), while 90% have a leg length difference of 1 mm or more (1). When the threshold of the leg length difference is set higher, a French epidemiological study revealed that approximately 1 in 1,000 individuals use a corrective orthopedic apparatus for LLD exceeding 2 cm (41). Often, compensatory strategies are used to protect the lumbar spine by functionally lengthening the shorter leg and shortening the longer leg during gait (42, 43), which may, in turn, result in long-term pathologies such as hip, knee, and lower back problems (40, 44, 45). In a recent case report, the development of synovial osteochondromatosis of the knee, complicated by LLD, was even reported (46). Some recent studies (47, 48) have reported compensation mechanisms in patients with mild LLD, defined as < 3 cm. Adaptations include a lateral tilt of the pelvis, counterbalanced by lowering the longer leg. These compensation strategies have been shown to correlate with LLD severity, suggesting that even mild forms may have negative long-term effects on the spine and the longer limb (48). Therefore, any asymmetries in the lower extremities as well as patients with LLD require a referral to an orthopedic specialist (49).

The etiology of LLD varies and can be categorized as either congenital or acquired. Examples of congenital LLD include hemiatrophy or hemihypertrophy (50) and fibular hemimelia (51), in which long bones do not form normally during pregnancy, leading to a discrepancy in leg length. Furthermore, foot deformities, such as equinovarus foot, may lead to the development of LLD (52). Acquired causes of LLD include idiopathic, trauma, infection, inflammation, and Legg-Calvé-Perthes (40). LLD can be as severe as the partial or complete absence of one limb. Furthermore, it can be present due to a shortening of one leg, which is more frequently the case, or due to a growth-promoting effect leading to a longer limb. Understanding the underlying etiology of LLD is important in determining which leg is pathological (4).

Severe LLD is commonly present in children with motor and muscle disorders, which lead to atypical bone development during the fetal period and also throughout childhood, as bone development and muscle activation are inextricably linked (53). Additionally, several studies in children with cerebral palsy also describe an association between bone deformities and atypical muscle tone (54–57).

To better understand and classify LLD, it is important to understand the normal regulation of lower extremity growth in humans. At skeletal maturity, the femur contributes approximately 54% to the total length of the lower extremity, whereas the tibia contributes 46% (58). The majority of the growth occurs around the knee (proximal tibia and distal femur growth plate), accounting for approximately 71% of the femur growth and 57% of the tibia growth (58).

## 5 Inflammation-induced LLD

**Figure 4:**
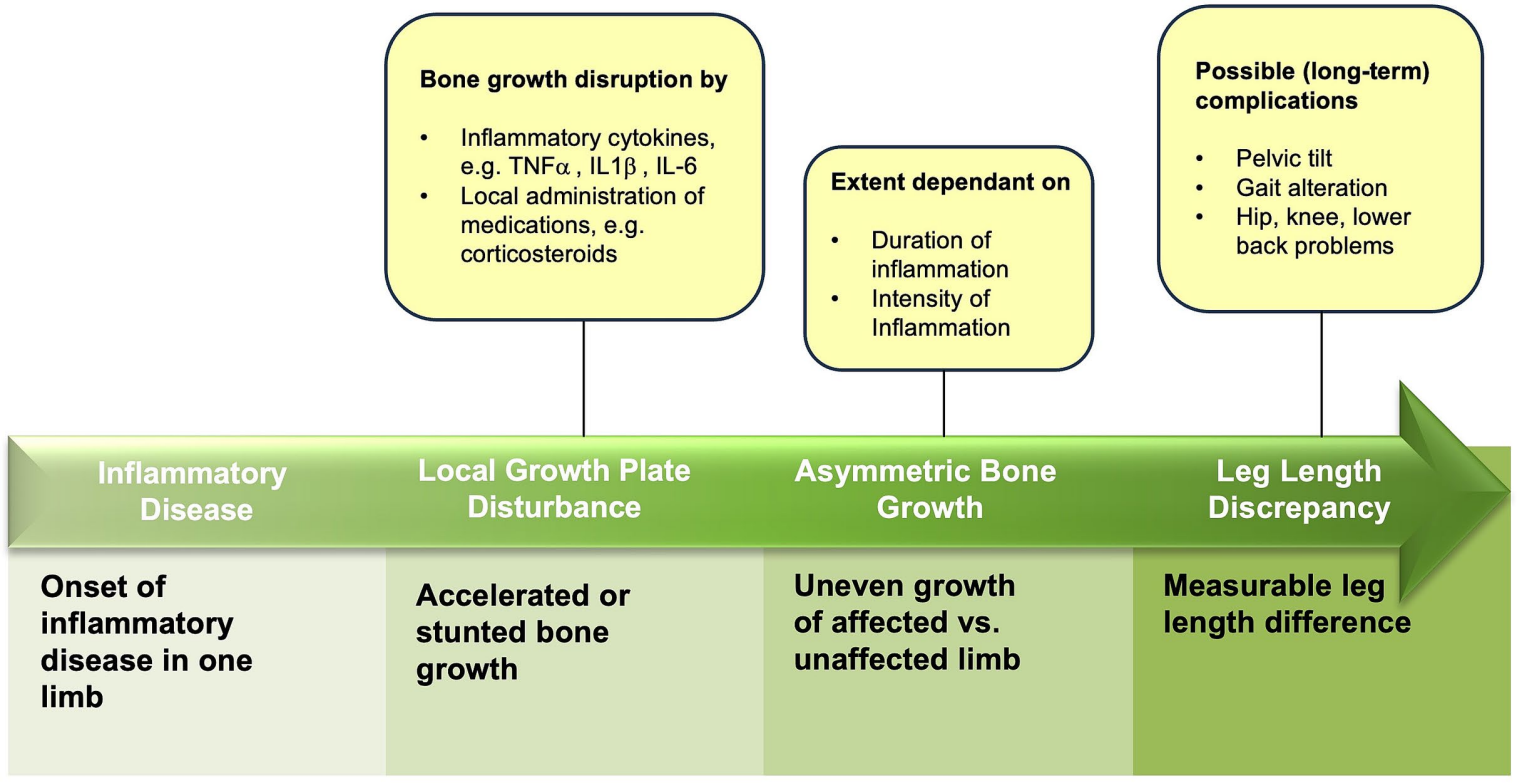
Overview of disease progress in inflammation-induced leg length discrepancy.

Longitudinal bone growth can either be promoted or inhibited by inflammation (10–13). An overview of all studies focusing on inflammation-induced LLD is provided in Supplementary Table 1, and the disease progression of inflammation-induced LLD is illustrated in Figure 4. As only a few studies are available to date, all of them were included in this review, although potential heterogeneity in study designs and patient populations exists between the studies (Supplementary Table 1). In addition, other factors, such as genetic background, nutrition, or concomitant medication (26), may also affect bone growth, therefore influencing the outcome. However, as most of these factors are systemic, both legs will be equally affected, thereby most likely not causing LLD.

### 5.1 Inflammatory diseases and their role in the development of LLD

Chronic inflammatory diseases, such as inflammatory bowel disease and some forms of JIA, are systemic inflammatory conditions. As a result, both legs and growth plates are equally exposed to inflammation. This, in turn, may not lead to LLD but rather to a general impairment of bone growth. Its degree varies from a mild decrease in growth velocity to severe short stature (12, 13). The pathogenesis of the growth-inhibiting effect is a combination of several factors, such as elevated levels of pro-inflammatory cytokines, malnutrition, hypercortisolism, and disease-related treatments (e.g., glucocorticoids), which may further inhibit bone growth (26).

However, when inflammation is present unilaterally in a leg or joint, it may lead to the development of LLD. Only a few inflammatory conditions are associated with LLD (Supplementary Table 1), including various subgroups of juvenile idiopathic arthritis (JIA), such as monoarthritic, oligoarticular polyarthritis, and pauciarticular JIA. In these conditions, arthritis is most often asymmetric, with the knee and/or ankle being the most frequently affected joints, leading to the development of LLD.

**Figure 5:**
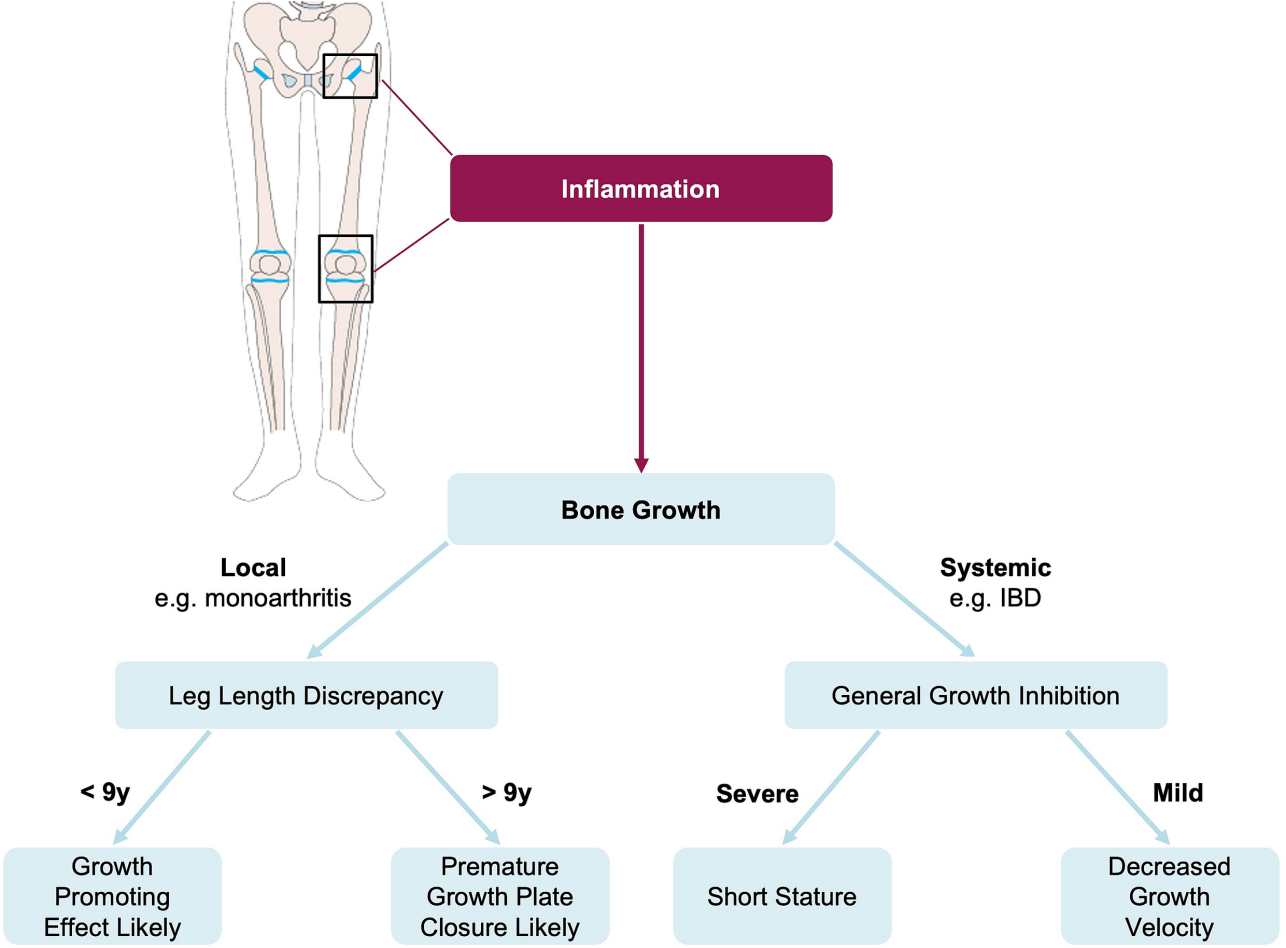
Effect of inflammation on bone growth and the development of leg length discrepancy. IBD = inflammatory bowel disease, y = years.

Interestingly, unilateral inflammation can result in either a growth-promoting or suppressive effect, which may lead to LLD (10, 11, 33, 34, 40, 59–63). Examples of conditions that may increase leg length include oligoarthritis and monoarticular/pauciarticular JIA (10, 34). Accelerated growth in the involved leg in monoarticular/pauciarticular JIA seems typical, as the ankle and knee are the most frequently affected joints, and arthritis is often asymmetric (60). We have created a flowchart to provide an overview illustrating the effects of inflammation on bone growth and the development of LLD (Figure 5).

### 5.2 Prevalence and extent of inflammation-induced LLD

The prevalence of LLD in children with JIA is largely unknown and depends on the specific condition, its treatment, and the availability of different treatment regimens. Therefore, making statements and comparisons is difficult. Overall, when considering all forms of JIA, LLD appears to develop relatively rarely, affecting approximately 5% of patients (64–66). However, when examining the different subgroups of JIA, the development of LLD seems most prevalent in oligoarthritic, monoarthritic, polyarthritic, and pauciarticular JIA.

In two cohort studies of patients with oligoarticular JIA (59, 67) the prevalence of LLD was found to be 22–24%. However, the degree of LLD was not stated; therefore, it is not clear whether significant LLD developed. In another study focusing on pauciarticular JIA with the involvement of a single knee, the prevalence of LLD was age-dependent. When the disease onset was below 3 years, 92% of patients with pauciarticular JIA developed LLD. In contrast, when the disease onset was after 3 years, only 47% showed an LLD (33).

In a study focusing on monoarthritic JIA with the involvement of a single knee, an LLD of up to 3.2 cm developed in two-thirds of the patients (68). However, this study was conducted in 1967 and used a treatment regimen that differs from current practices.

Furthermore, the prevalence of LLD in children with JIA receiving different treatments has been investigated. One study (60) tested the use of intraarticular steroids in patients with pauciarticular JIA and found that it could prevent the development of LLD. Specifically, no patients developed LLD in the treatment group, whereas in the untreated control group, 50% were found to have an LLD. However, a published systematic review found weak and overall inconclusive evidence for a decrease in the development of LLD when intraarticular steroids were used (62). In addition, the use of methotrexate and biologics has been compared in terms of acquiring an LLD (69). In oligoarthritis, 11% of patients treated with biologics developed LLD compared to 8.8% who were treated with methotrexate. This finding is in contrast to patients with polyarthritis, where an opposite effect was observed, with 6.6% of the patients in the methotrexate group who developed LLD compared to 4.5% with biologics.

### 5.3 Age of disease onset, course, and dynamics of inflammation-induced LLD

Simon et al. (34) show an association between the age of monoarticular and pauciarticular JIA onset and the effect of inflammation on leg growth. When unilateral disease was present in patients with an onset of the disease below 9 years of age, 97% showed a growth acceleration on the pathological side. In contrast, when the disease onset was >9 years, a growth arrest due to a premature closure of the growth plate of the involved side was present in 80%, leading to a shortening of the affected leg.

In another study (33), all patients with pauciarticular arthritis showed a growth-promoting effect on the affected leg [mean age of onset of the two groups (>3y and <3y): 1.7 and 7 years; *n* = 32]. Unfortunately, only the mean age of the two comparison groups is provided, not the age range. In all other studies, it remains unclear whether growth acceleration or inhibition was observed, as they only report leg length discrepancy without specifying the actual effect on the affected leg. Therefore, making a definitive statement regarding the relationship between the age of onset and the effect of inflammation on bone growth is difficult, although an age-dependent effect seems plausible.

Regardless of the age of onset of inflammation, the major leg length discrepancy normally develops within the first 4 years after disease onset (34). Thereafter, a different dynamic between individuals could be observed so that LLD either remained at level, decreased, or slowly increased, except for a rapid, premature growth plate closure. Interestingly, in some patients, LLD decreased so that there was a gradual inhibition of growth in the pathological leg (33% of patients) (34). In patients diagnosed at an early age (<3 years), an association between the duration of the first episode of joint swelling and the development of LLD could be found (33).

Few studies are available in the literature assessing the extent of inflammation-induced LLD. When an acceleration of growth was present in the affected leg, the LLD described in published papers did not exceed 3.5 cm, indifferent of JIA subgroup (33, 34, 60, 63, 68, 70). It is not surprising that LLD was greater in patients where a premature growth plate fusion occurred, reaching up to 5.9 cm (34).

### 5.4 General treatment of LLD

Available treatment options for LLD, irrespective of its etiology, include non-surgical and surgical treatment. The treatment choice depends on various factors such as the degree of LLD, age of the child, and severity of symptoms.

**Figure 6:**
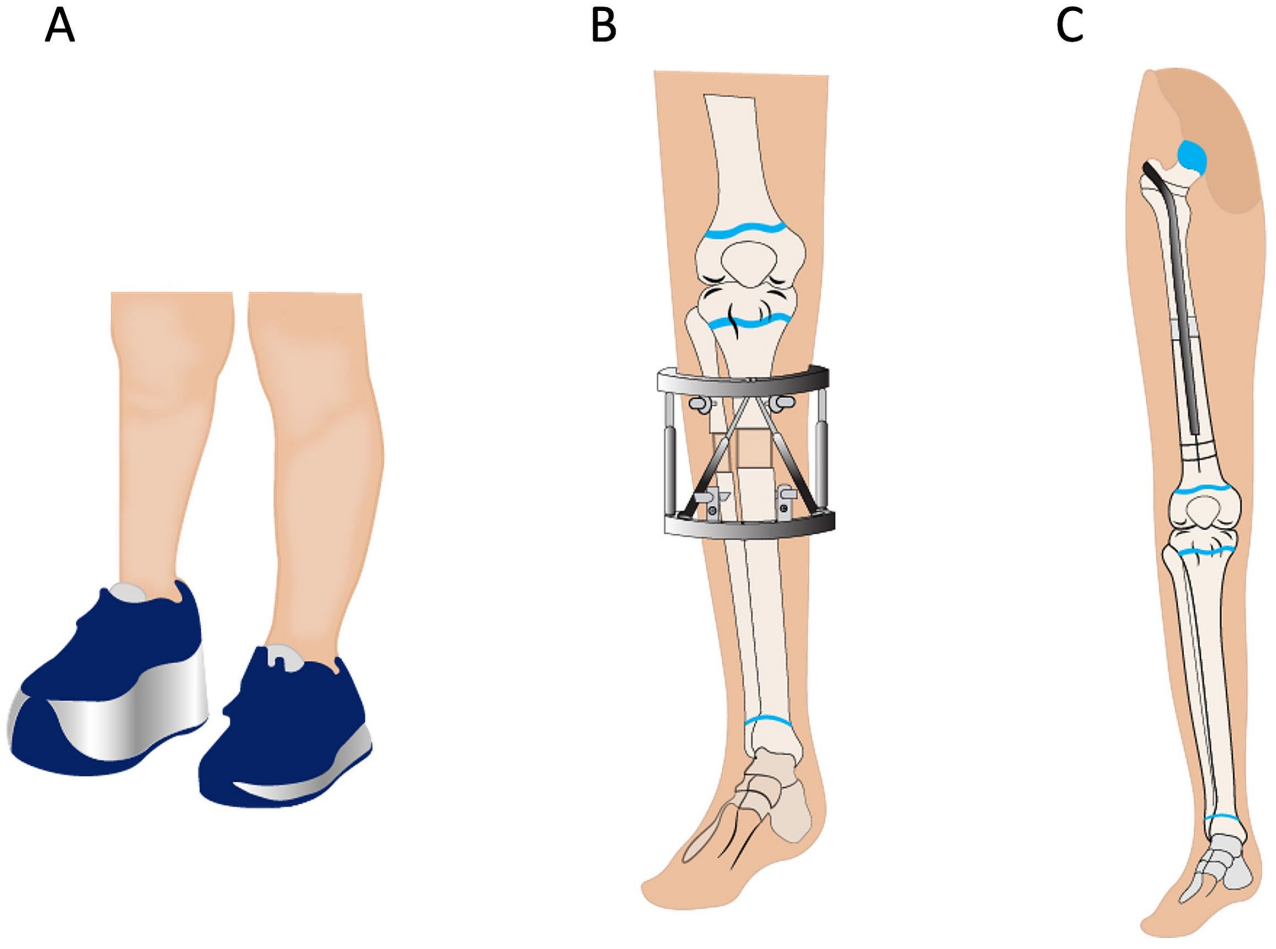
Different treatment options in patients with leg length discrepancy. **(A)** External shoe lift. **(B)** Leg lengthening by the Ilizarov procedure. **(C)** Intramedullary lengthening nail.

Table 1 provides an overview of different treatment options depending on the extent of LLD. When it comes to the treatment of LLD, different treatment options should be considered on an individual basis, with careful consideration of the risks and benefits. An LLD less than 2 cm is usually not treated (45). However, internal shoe lifts may be used when the LLD ranges from 0.5 to 1.5 cm, while patients are more comfortable with external shoe lifts when the LLD is between 1.5 and 2 cm (71) (Figure 6A). Physical therapy, such as stretching the muscles of the lower extremity, is also a non-surgical treatment option for LLD, but it is only used for functional scoliosis. There, the LLD is due to pelvic obliquity from adaptive soft-tissue changes, muscle contracture, or ligamentous laxity (71). When an LLD exceeds 2 cm, surgical treatment, for examples, leg shortening or lengthening may be considered. However, conservative treatment with a shoe lift is a valuable alternative, where the LLD may be corrected to 1–2 cm residual inequality (4, 72). In a patient with an LLD between 2 and 5 cm, open or closed epiphysiodesis as well as stapling before skeletal maturity exist as possible treatment options. However, surgical treatment requires the exact timing of surgery and complications after epiphysiodesis, such as joint pain, knee stiffness, angular deformities, and altered proximal tibial articular surface, may develop (73, 74).

**Table 1:** Different treatment options depending on the extent of leg length discrepancy.

Extent of leg length discrepancy	Treatment
< 2 cm	Usually, no treatment is required Between 0.5 and 1.5 cm: internal shoe lifts 1.5 and 2 cm: external shoe lifts
2–5 cm	Conservative Epiphysiodesis (open or closed) Physeal stapling Shortening osteotomy
6–10 cm	Consider leg lengthening
> 15 cm	Lengthening of the shorter leg and shortening of the longer leg
> 20 cm	Usually treated with prostheses

For an LLD ranging between 6 and 10 cm, leg lengthening of the shorter leg should be considered. The traditional Ilizarov procedure (Figure 6B) (75) using callus distraction histogenesis is the mainstay of all new lengthening treatment modalities used in recent times. When using an external fixator, possible complications include skin infection, pain, delayed bone union or non-union, joint stiffness, and deformities. The preference is currently shifting toward motorized intramedullary lengthening nails (Figure 6C), which do not require rotation for distraction (76). With the intramedullary nails, stiffness of the surrounding joints may still occur, but skin infections are rare.

An LLD greater than 15 cm usually requires lengthening of the shorter leg combined with shortening of the longer leg. An internal or external lift may also be used if equalization is not achieved postoperatively. An LLD of more than 20 cm is usually treated with prostheses (77).

### 5.5 Treatment of inflammation-induced LLD

Few studies are available in the literature specifically addressing the treatment of inflammation-induced LLD. In Finnish studies, Skyttä et al. (63, 70) investigated the long-term results of temporary epiphyseal stapling in patients who developed LLD due to JIA. The indication for the procedure was an LLD exceeding 10 to 15 mm (depending on the age and therefore growth potential of the child). The mean age at the time of surgery was 11 years, and the mean duration of the disease was 7 years. The procedure was quite effective, reducing the median leg length discrepancy before surgery from 17.5 mm to 5 mm by the time of the removal of the staples. The method had a complication rate of 10%, and complications included peroneal paralysis, infection, premature loosening, physeal plate perforation, breakage of the staple, and mislocation (63).

## 6 Conclusion

Patients with monoarticular, pauciarticular, or polyarthritic JIA are at risk of developing LLD when inflammation is predominantly present in one limb. Inflammation may lead to the acceleration or inhibition of bone growth in the affected leg, most likely depending on the age of onset. The LLD extent ranges from mild forms, where treatment is not necessary, to severe forms, leading to premature growth plate fusion and LLD of 5 cm or more. It is, therefore, important to identify patients at risk of developing LLD early, enabling early treatment initiation.

## Footnotes

^1^Available at www.covidence.org
