# Supplementary material for: Inflammation-induced leg length discrepancy in children: from molecular mechanisms to clinical implications
Source: Front Med (Lausanne). 2025 May 20;12:1542822. doi: 10.3389/fmed.2025.1542822 (PMC12129924; doi:10.3389/fmed.2025.1542822)
Supplement: Supplementary file 1 [file Table_1.docx]

**Supplementary Table 1. Summary of studies on inflammation-induced leg length discrepancy**

| **Author (year)** | **Study design** | **Disease** | **Study population and size** | **Cohort size** | **Age** | **Treatment** | **LLD extent/ prevalence** | **Effect on bone growth** | **Disease course** |
| --- | --- | --- | --- | --- | --- | --- | --- | --- | --- |
| Baris (2018) | Retrospective cohort study | JIA | Boston Children´s Hospital (MA, USA)  *n*=76 | *n*=76 | Mean age: 4.3y (range 2.3-7.2y) | Biologics (60%), steroids | 5% of all patients developed LLD (unclear how much LLD in cm) in biologic disease modifying antirheumatic drugs | NA | NA |
| Cassidy JT (1967) | unclear | Monoarticular juvenile rheumatoid arthritis | Study population unclear  *n*=40 | *n*=40 |  |  | LLD up to 3.2 cm, seen in 2/3 of patients with involvement of a single knee | NA | NA |
| Chhabra (2020) | Prospective cohort study | JIA (oligoarthritis, ERA, RF-neg/pos polyarthritis, systemic, psoriatic, undifferentiated) | British Columbia Children´s Hospital in Vancouver and McMaster Children´s Hospital in Hamilton (Canada)  *n*=247 | *n*=247 | 10.6y (range 4.8-13.3y) | Biologics, NSAID, joint injection | 5% of all patients showed LLD (not differentiated) | NA | NA |
| De Oliveira Sato (2011) | Cross-sectional and retrospective analysis | Oligoarticular JIA | Paediatric rheumatology clinic (unclear where)  *n*=75 | *n*=75 | Age at disease onset 7.1y (range 3.6-9.2) | NSAID (97%), Prednisone (7%), steroid injection (52%), DMARD (25%). | 22.7% of all patients developed LLD | NA | NA |
| Giancane (2019) | Retrospective cohort study and cross-sectional | Oligoarthritis and polyarthritis JIA | Paediatric rheumatology units of Istituto G. Gaslini of Genoa and Fondazione Policlinico S. Matteo of Pavia (Italy)  *n*=239 (methotrexate)  *n*=269 (biologic era) | *n*=239 (methotrexate)  *n*=269 (biologic era) | 3.1y (range 1.8-5.8y) (methotrexate)  2.5y (1.6-4.7y) in biologic era. | Methotrexate, biologics, systemic or intraarticular steroids | Oligoarthritis:  -methotrexate treated: *n=*9 (8.8%)  -biologic era: *n=*15(11%)  Polyarthritis:  -methotrexate treated: *n=*9(6.6%)  -biologic: *n=*6(4.5%) | NA | NA |
| Moued (2013) | Retrospective cohort study | Oligoarticular JIA | Children followed at King Abdulaziz University Hospital, Jeddah, Saudi Arabia)  *n*=37 | *n*=37 | Mean age at disease: 7.2y | Naproxen (100%), intra-articular steroids (32%), methotrexate (38%), adalimumab (13%) | 24% of patients developed LLD | NA | NA |
| Sherry (1999) | Retrospective cohort study | Pauciarticular juvenile rheumatoid arthritis | Children from Seattle (WA, USA) and in Chapel Hill and Durham (NC, USA)  *n*=30 | *n*=30 | <7y | Intraarticular steroid injection (one group) vs. no injection (2^nd^ group) | 7 (untreated) vs. 0 (steroid), range: 0.5-3.5cm. Mean: 1.0±1.4cm | Promotion | NA |
| Simon (1981) | Retrospective cohort study | Monoarticular and pauciarticular Juvenile Rheumatoid Arthritis | Children referred to the Growth Study Clinic at the Children´s Hospital Medical Center, Boston (MA, USA)  *n*=100 | *n*=100 | 72/100 patients, disease occurred <5y | Acetylsalicylic acid,  2 patients treated with steroids | <9y: overgrowth  >9y: 4/5 rapid premature closure of growth plate  Range LLD: 0.9-5.9cm | Promotion (mostly <9y), inhibition (mostly >9y) | Increased very slowly after 3-4 y, remained level or even decreased |
| Skyttä (2003) | Retrospective cohort study | Systemic arthritis, polyarthritis, oligoarthritis, psoriatic arthritis, other | Finnish children treated at Rheumatism Foundation Hospital (Heinola, Finland)  *n*=71  Systemic arthritis (2%), polyarthritis (29%), oligoarthritis (63%), psoriatic arthritis (3%), other (3%) | *n*=71  Systemic arthritis (2%), polyarthritis (29%), oligoarthritis (63%), psoriatic arthritis (3%), other (3%) | Mean time of OP 11y (range 5-16), mean duration of disease 7y (range 1-14y) | Temporary epiphyseal stapling;  some with synovectomies, arthrodesis, arthroplasties and intra-articular steroid injections | Before OP: 17.5mm (range approx. 3mm-34.5mm) | NA | Unclear, only patients referred for OP |
| Skyttä (2003) | Retrospective cohort study | Systemic arthritis, polyarthritis, oligoarthritis | Finnish children treated at Rheumatism Foundation Hospital (Heinola, Finland)  *n*=17  Systemic arthritis (1), polyarthritis (4), oligoarthritis (12) | *n*=17  Systemic arthritis (1), polyarthritis (4), oligoarthritis (12) | Range 6-15y at time of OP (mean duration of disease at time of OP 7y) | Temporary epiphyseal stapling;  some with synovectomies, arthrodesis, arthroplasties and intra-articular steroid injections | Before OP: mean LLD was 21mm (approx. 7mm-33mm) | Promotion (not explicitly mentioned) | Unclear, only patients referred for OP |
| Tangcheewinsirikul  (2023) | Cross-sectional study | ERA, systemic JIA, Poly-RF+ and -, | Paediatric Rheumatology Clinic, Faculty of Medicine Siriraj Hospital, Mahidol University, Bangkok (Thailand)  *n=*101 | *n=*101 | IQR: 13.8-9.9y | NSAIDs, corticosteroids, biologic agents | NA | NA | NA |
| Vostrejs (1988) | Prospective cohort study | Pauciarticular juvenile rheumatoid arthritis | Children from Denver (CO, USA)  *n*=32 | *n*=32 | 2 groups:  Mean 1.7y (<3y, *n*=13), mean 7y (>3y, *n*=19) | Aspirin | 1.4±0.2cm (<3y; range 0 to 2.5cm); 0.6±0.2cm (>3y; range 0 to 2cm) | Promotion | NA |

Abbreviations: DMARD=disease modifying anti-rheumatic drug, ERA=enthesitis-related arthritis, IQR=interquartile range, JIA=juvenile idiopathic arthritis, LLD=leg length discrepancy, NA=not available, NSAID=non-steroidal anti-inflammatory drug, OP=operation, Poly +/-=polyarthritis RF positive/negative, RF=rheumafactor, y=years
